# Progress towards health equity in Vietnam: evidence from nationwide official health statistics, 2010-2020

**DOI:** 10.1136/bmjgh-2023-014739

**Published:** 2024-03-18

**Authors:** Yikai Feng, Tran Diep Tuan, Junyi Shi, Zhuo Li, Mailikezhati Maimaitiming, Yinzi Jin, Zhijie Zheng

**Affiliations:** 1 Department of Global Health, School of Public Health, Peking University, Beijing, China; 2 Institute for Global Health and Development, Peking University, Beijing, China; 3 University of Medicine and Pharmacy, Ho Chi Minh City, Viet Nam; 4 Institute of Area Studies, Peking University, Beijing, China

**Keywords:** health policy

## Abstract

**Introduction:**

One of the ultimate goals of strengthening the health system is to achieve health equity. Vietnam is considered one of the ‘fast-track countries’ to achieve the health-related Millennium Development Goals, but research on its equity strategies remains inadequate.

**Methods:**

Using Vietnamese official health statistics, we investigated inequity in four dimensions including health resources, service delivery, service utilisation and residents’ health status from the perspectives of income levels, poverty rates and subnational regions. The Slope Index of Inequality, concentration curve/Concentration Index, absolute difference and Theil Index were used.

**Results:**

Four indicators showed ‘pro-poor’ inequality in health resources, including the per capita health budget, per capita health personnel, per capita health personnel at the community level and per capita hospital beds at the community level, while provincial hospital beds showed ‘pro-rich’ inequality. Two health service delivery indicators (delivery of antenatal care ≥3 times and proportion of community health service centres with medical doctors) show ‘pro-rich’ inequality, although two health status indicators, mortality and malnutrition rates for children under five, showed ‘pro-poor’ inequality. The Northern Midlands and Mountain Areas, and the Central Highlands were disadvantaged regarding service delivery and health status. Intraregional differences were the main factors contributing to the inequalities in delivery of antenatal care ≥3 times, provincial hospital beds and percentage of community health centres with medical doctors, with the Red River Delta and the South East region experiencing the greatest inequalities.

**Conclusion:**

The overall level of health equity in Vietnam has increased over the past decade, although inequality in health service delivery has hindered progress towards health equity based on income, poverty and subnational regions. Targeted policies need to be introduced to reduce inequities relating to the health workforce and service delivery capacity.

**WHAT IS ALREADY KNOWN ON THIS TOPIC**

- Vietnam’s health services coverage is at the forefront of Association of Southeast Asian Nations member states, and its effort to achieve equity has made considerable progress in recent years.
- The Vietnamese government is committed to ongoing health equity improvement but the process of this task is not without obstacles.
- Few studies have focused on the development of health equity in Vietnam from the health-system perspective, nor from the perspectives of income/wealth and subnational regions.

**WHAT THIS STUDY ADDS**

- In this study, we used official yearbook data to describe the development of health equity in Vietnam from 2010 to 2020 from a health-system perspective.
- Inequities remain between affluent and poor areas in relation to the allocation of professional health workers and the provision of primary healthcare services.
- Health status varies among regions, and there are also variations among provinces within relatively developed regions.

**HOW THIS STUDY MIGHT AFFECT RESEARCH, PRACTICE OR POLICY**

- Our results provide guidance for the future development of the health system in Vietnam and other developing countries.
- While it is necessary to strengthen investment in key areas and improve hardware, it is also important to focus on efficiency, strengthen service capabilities and adjust policies based on health outcomes.
- Vietnam’s health policies focused on the poor provide a lesson for other lower-middle-income countries, as do the challenges faced by Vietnam in developing greater health equity.

## Introduction

Health equity is the key to achieving universal health coverage (UHC).^1 2^ Health inequities are the unjust differences in health between persons of different social groups and can be related to different forms of disadvantage such as poverty.^3^ Disadvantaged regions with marked inequalities can lack the resources necessary to participate in society’s socioeconomic mainstream, thereby increasing the existing gaps.^4^ Introducing health equity reforms typically involves advocating on behalf of society’s poor and marginalised, who might lack effective political representation.^5^ Targeting policies and resources towards subregions with the greatest need could help to narrow these gaps and contribute to the achievement of development goals.^6^ Thus, it is important to understand the lessons learnt at the national level when formulating targeted policies and cooperative programmes in this field.

Vietnam has a stable natural, political and economic environment, and is considered one of the 10 ‘fast-track countries’ in terms of achieving the health-related Millennium Development Goals.^7^ Its UHC Service Coverage Index value reached 70 in 2021,^8^ which is relatively high among Association of Southeast Asian Nations (ASEAN) countries (after Singapore, Brunei and Thailand). Vietnam has also made significant progress towards achieving health equity.^9 10^ Researchers found that though there is a reduction of health inequity in most countries, inequity persists in some countries among ASEAN. For instance, Cambodia, Indonesia, Lao PDR and Timor-Leste are faced with pro-specific regions. Vietnam’s health services coverage is at the forefront of ASEAN member states, and its effort to achieve equity has also made considerable progress in recent years.^9^

The Vietnamese government is committed to ongoing improvement. As the Deputy Director of the Ministry of Health’s Department of Planning and Finance noted, ‘Vietnam has strongly emphasized the importance of equity in health with a series of pro-poor health policies, seeking to achieve small gaps in health outcomes between the poor and better off’.^11^ Vietnam’s pro-poor policies and measures have played an important role in reducing the economic burden on patients and reduced catastrophic health expenditures.^12 13^ One of the most commendable policies targeting the poor is Vietnam’s social health insurance (SHI) programme. In 2008, the Vietnamese government passed legislation to integrate the Health Care Fund for the poor, ethnic minorities and vulnerable groups into the SHI. SHI’s coverage increased to 90.9% in 2020, and the current programme covers a comprehensive range of benefits, including emergency services, Medical care (inpatient and outpatient), medication and medical supplies.^14^ In a series of healthcare policies since the 1980s, inequity alleviation has become a key task targeting poverty-stricken areas. The *Plan for People’s Health Protection, Care, and Promotion in 2016–2020* prioritised the development of health services in impoverished mountainous and remote areas and provided medical and health benefits to impoverished people and the beneficiaries of social policies. Specific measures include a series of health workforce development projects targeting impoverished areas, especially the development of primary healthcare practitioners.^15^ However, the process of promoting health equity in Vietnam is not without obstacles. A report from the World Bank states that resource flows and policies still favour tertiary care in developed areas of Vietnam, and the health system remains strongly hospital-centric.^16^ Researchers also pointed out that numerous health plans and strategies have proposed a series of ambitious development goals, but failed to formulate strategic measures targeted towards specific situations in different regions.^17^

As one of the lower-middle-income countries (LMICs),^18^ Vietnam provides an ideal opportunity for in-depth research on its health development-related strategies, achievements and challenges to provide guidance for other countries with similar economic conditions. Some previous studies have examined health equity in Vietnam, mainly focusing on areas such as maternal and child health, and non-communicable diseases. For example, Nguyen *et al* used Bayesian modelling to analyse trends in equity regarding reproductive, maternal, newborn and child health service coverage in Vietnam from 2000 to 2030, confirming Vietnam’s positive momentum.^19^ Le *et al* examined a range of socioeconomic factors contributing to health inequity in relation to non-communicable diseases among elderly people in Vietnam,^20^ while Baek *et al* examined the equity impacts of a multicomponent early childhood development intervention in rural Vietnam.^21^ No studies have focused on the development of health equity in Vietnam from the health-system perspective, nor from the perspectives of income/wealth and subnational regions.^22^ This has resulted in significant difficulties in identifying specific challenges in relation to achieving UHC.

It is worth noting that sources of microdata for equity research in Vietnam are relatively limited. In this study, we used official yearbook data to describe the development of health equity in Vietnam from 2010 to 2020 from a health-system perspective. We identified various development bottlenecks, and thus our results provide guidance for the future development of the health system in Vietnam and other developing countries.

## Methods

### Data source

Vietnam comprises 63 provincial-level divisions (58 provinces and five municipalities). These divisions are divided into six socioeconomic subregions, including the Central Highlands, Mekong River Delta, North Central and Central Coastal Areas, Red River Delta, South East, and Northern Midlands and Mountain Areas. Geographically, 64% of the impoverished population lives in the Northern Midlands and Mountain Areas, the Central Highlands, and the Northern Central and Central Coastal Areas.^23^

We used panel data for 63 provincial-level divisions between 2010 and 2020 from the *Vietnam Health Statistics Yearbook* and *Statistical Yearbook of Vietnam*. The *Vietnam Health Statistics Yearbook* is an annual report that contains administrative health data reported to the Vietnam Ministry of Health by various departments, ministries, health programmes and institutes.^24^ The *Statistical Yearbook of Vietnam* is an annual publication by the Vietnam General Statistics Office, comprises basic data reflecting the general socioeconomic dynamic and situation of the whole country, socioeconomic regions, and localities.^25^ We selected 12 indicators to measure the equity of Vietnam’s health system in four dimensions:^26 27^ health resources, health service delivery, health service utilisation and residents’ health status (see online supplemental appendix table A1). The socioeconomic indicators include population, average monthly income and incidence of poverty.

10.1136/bmjgh-2023-014739.supp1 Supplementary data

### Data analysis

We used the Slope Index of Inequality (SII) and the concentration curve/Concentration Index to analyse inequality based on income and the incidence of poverty, and the absolute difference and the Theil Index (TI) to analyse inequality based on subnational regions.

The SII measures the absolute inequality between groups in a natural order, and evaluates differences between regions.^28 29^ To calculate the SII, we calculated the health system indicator value of each provincial-level division and ranked these divisions based on their socioeconomic status. Regression analysis was used to obtain the fitting line, the slope of which is the SII. The formula is as follows:

(1) $$Y_{j}=\beta_{0}+\beta_{1}R_{j}$$

where *Y_j_* is the health system indicator value for province *j* and *R_j_* is the population-weighted correlation rank for province j with a value range of (0,1). To ensure the robustness of the results, we used both per capita income and incidence of poverty in ascending order as ranking criteria. When the ranking is based on per capita income, if the SII value is greater than 0, it is considered ‘pro-rich’ inequality, and vice versa. When the ranking is based on the incidence of poverty, if the SII value is greater than 0, it is considered ‘pro-poor’ inequality, and vice versa.

The concentration curve is used to represent the relative inequality between groups in natural order.^30 31^ The independent variable is the cumulative percentage of regions sorted by economic level, with a value range of (0,1). The dependent variable is the cumulative percentage of observed indicators for the corresponding provinces, with a value range of (0,1). If the health status of provinces with different economic levels is equal, the concentration curve is on the diagonal. If the health indicators in low-income provinces have lower values, the concentration curve is located below the diagonal, and if they have higher values, the concentration curve is located above the diagonal. The further the curve is from the diagonal, the more unequal the values. The Concentration Index is a quantification of the concentration curve, the absolute value of which is twice the area between the concentration curve and the diagonal, with the Concentration Index values in a range of (–1,1). If the health status related to the economic level is equal, the Concentration Index value is 0. When the concentration curve is above the diagonal, the Concentration Index value is negative, indicating that the indicator is more inclined towards impoverished provinces. When the concentration curve is below the diagonal, the Concentration Index value is positive, indicating that the indicator is more inclined towards affluent provinces. The Concentration Index formula is as follows:

(2) $$CI=2\times \frac{cov(h,r)}{\mu }$$

where *h* is the health indicator value for each province, *μ* is the average for each province, and *r* is the cumulative percentage of provinces sorted by per capita income or incidence of poverty.

Tools provided by the *International Center for Equity in Health* were used to analyse the development trends of absolute health differences at the regional level and to identify vulnerable areas,^32 33^ and the TI was used to find regions with significant internal differences. The TI describes inequalities that do not exist in natural order groups and can be decomposed into different regions by measuring intragroup and intergroup differences and their respective contributions to the overall difference.^34^ Overall differences can be decomposed into intragroup and intergroup differences, as shown below:

(3) $$I_{t}=I_{w}+I_{b}=\sum_{g=1}^{G}X_{g}\left[\sum_{i\epsilon S_{g}}Log\left(\frac{\frac{x_{i}}{X_{g}}}{\frac{1}{N_{g}}}\right)\right]+\sum_{g=1}^{G}X_{g}Log\left(\frac{X_{g}}{\frac{N_{g}}{N}}\right)$$

where $I_{w}$ is the intergroup difference, $I_{b}$ is the intragroup difference, G is 6, representing the number of Vietnam’s socioeconomic subregions,^23^ $N_{g}$ is the number of provinces in the g^th^ region, $x_{i}$ is the proportion of the absolute value of the health indicators of province *i* in the region and $X_{g}$ is the proportion of health indicator values in the g^th^ region at the national level.

## Results

### Overall development

**Figure 1:**
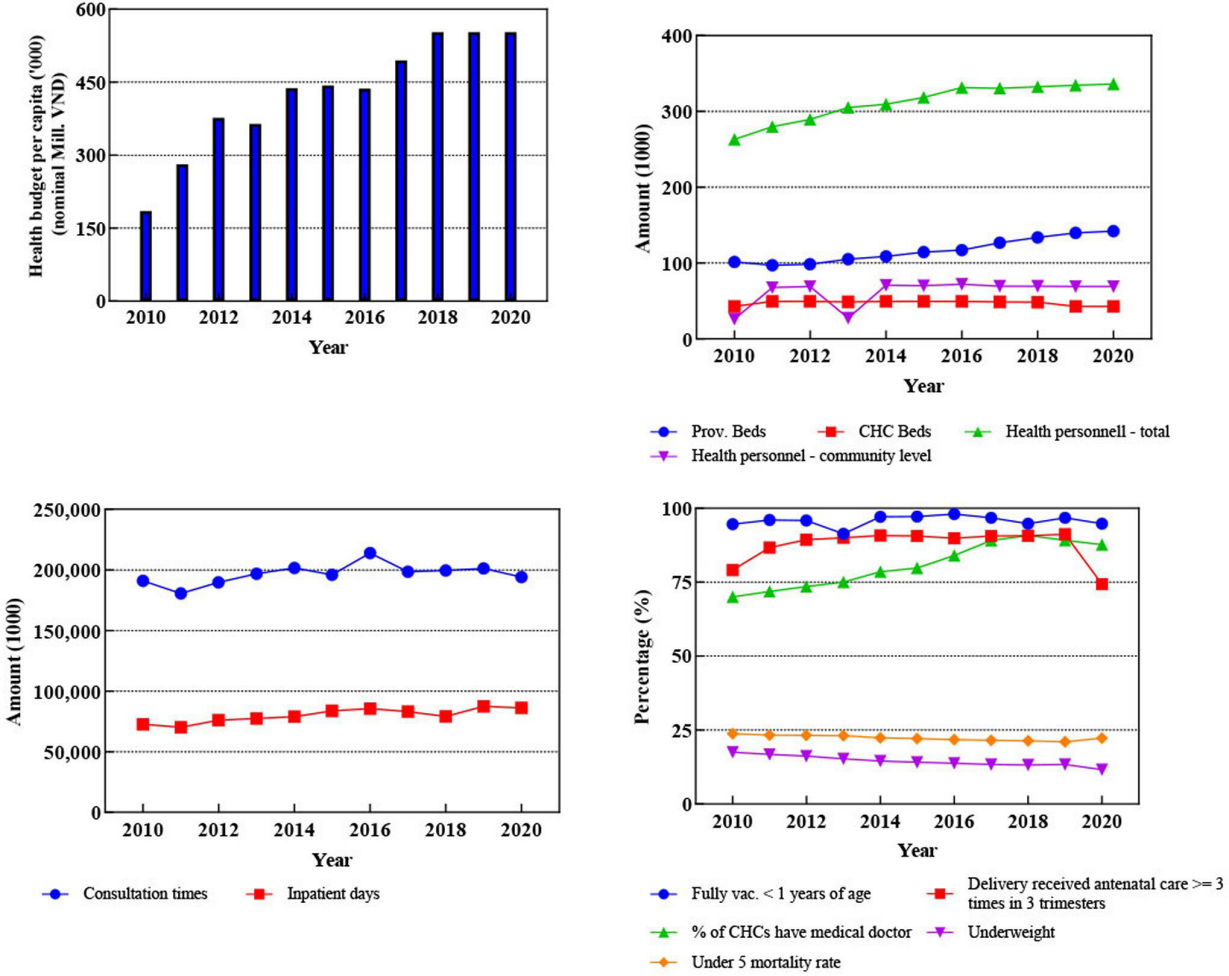
Trends in health system indicators in Vietnam (2010–2020).

Vietnam made progress in all four dimensions during the period from 2010 to 2020. In terms of health resources, Vietnam’s health budget per thousand people increased by 198.4% from 185.3 million VND (Vietnamese Dong) to 552.9 million VND (nominal). The health workforce also increased significantly, from 263 256 to 336 252 people, while the number of provincial hospital beds increased from 101 573 to 142 229. Improvements have occurred in relation to primary health construction, with the proportion of community health centres with doctors increasing from 70% to 87.7%. In terms of health services utilisation, the total number of inpatient days increased by 20%. In terms of health status, the proportion of children under 5 years of age suffering from malnutrition decreased from 17.5% in 2010 to 11.6% in 2020. Although delivery of antenatal care ≥3 times had remained stable at around 90% for many years, in 2020 it fell to 74.4%. The changes in other health indicators are shown in figure 1 (detailed data and trend test results are shown in online supplemental appendix table A2).

### Equity status based on income and the incidence of poverty

**Figure 2:**
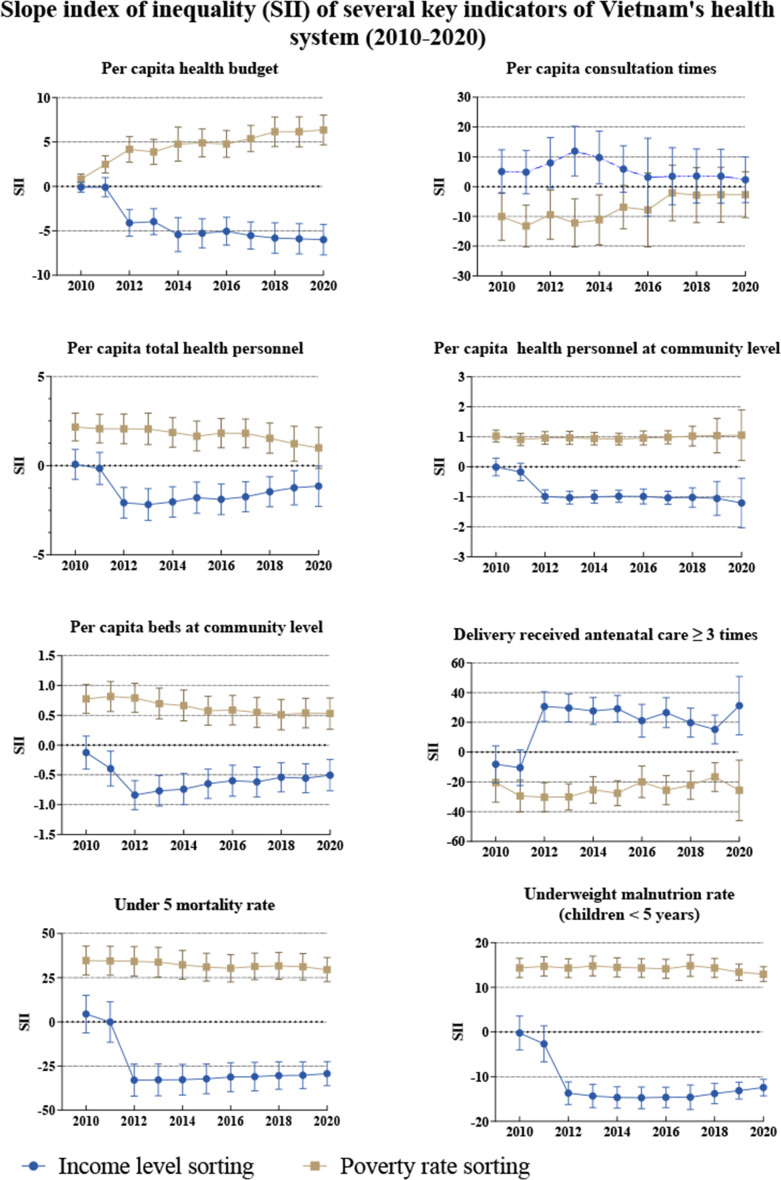
Trends in SII values for several key indicators of Vietnam’s health system (2010–2020).

The trends in the SII values for the selected health system indicators from 2010 to 2020 are shown in figure 2 (see online supplemental appendix tables A3 and A4 for the detailed data and trend testing results). Four indicators including per capita health budget, per capita health personnel, per capita health personnel at the community level and per capita hospital beds at the community level showed ‘pro-poor’ inequality (SII-Income sorting<0, SII-Poverty rate sorting>0). The results of trend testing showed that the SII value for the per capita health budget and per capita hospital beds at the community level displayed a significant linear trend (p<0.05), indicating that ‘pro-poor’ inequality is increasing. The SII values for per capita consultation times and inpatient days were both close to 0, indicating that affluent and poor provinces tend to be similar in terms of health service utilisation. In terms of full immunisation among children, both SII values showed no statistical significance in recent years. In terms of delivery of antenatal care ≥3 times, Vietnam has long displayed ‘pro-rich’ inequality (SII-Income sorting>0, SII-Poverty rate sorting<0), and this increased after 2019. The mortality and malnutrition rates for children under the age of 5 years showed significant ‘pro-poor’ inequality (SII-Income sorting<0, SII-Poverty rate sorting>0).

**Figure 3:**
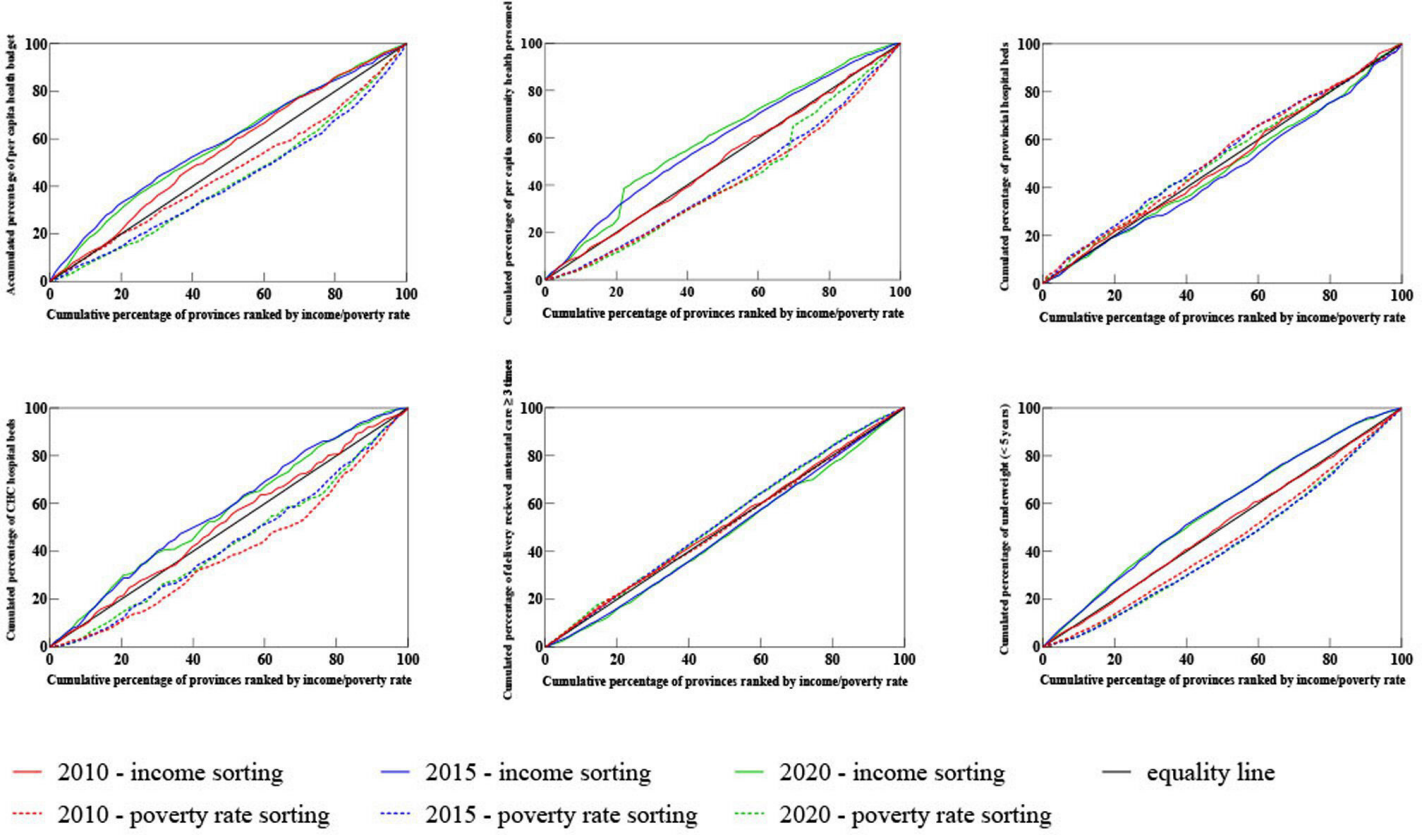
Concentration curves for several key indicators of Vietnam’s health system (2010, 2015 and 2020).

The Concentration Indexes for the selected indicators in 2010, 2015 and 2020 are shown in figure 3 (detailed data and trend testing results are shown in online supplemental appendix tables A4 and A5). During 2010 and 2020, four indicators including health budget, per capita health personnel at community level, per capita hospital beds at the community level showed ‘pro-poor’ inequality (Concentration Index-Income sorting<0, Concentration Index-Poverty rate sorting>0). The results of trend testing showed that the Concentration Index for the per capita health budget, per capita hospital beds at the community level and per capita health personnel at the community level displayed a significant linear trend (p<0.05), indicating increasing ‘pro-poor’ inequality. The opposite applied in relation to the delivery of antenatal care ≥3 times and the proportion of community health service centres with medical doctors (Concentration Index-Income sorting>0, Concentration Index-Poverty rate sorting<0). Regarding the mortality and malnutrition rates in children under 5 years of age, there was significant inequality between affluent and poor areas, with higher incidences in poor areas (Concentration Index-Income sorting<0, Concentration Index-Poverty rate sorting>0).

### Equity based on subnational regions

**Figure 4:**
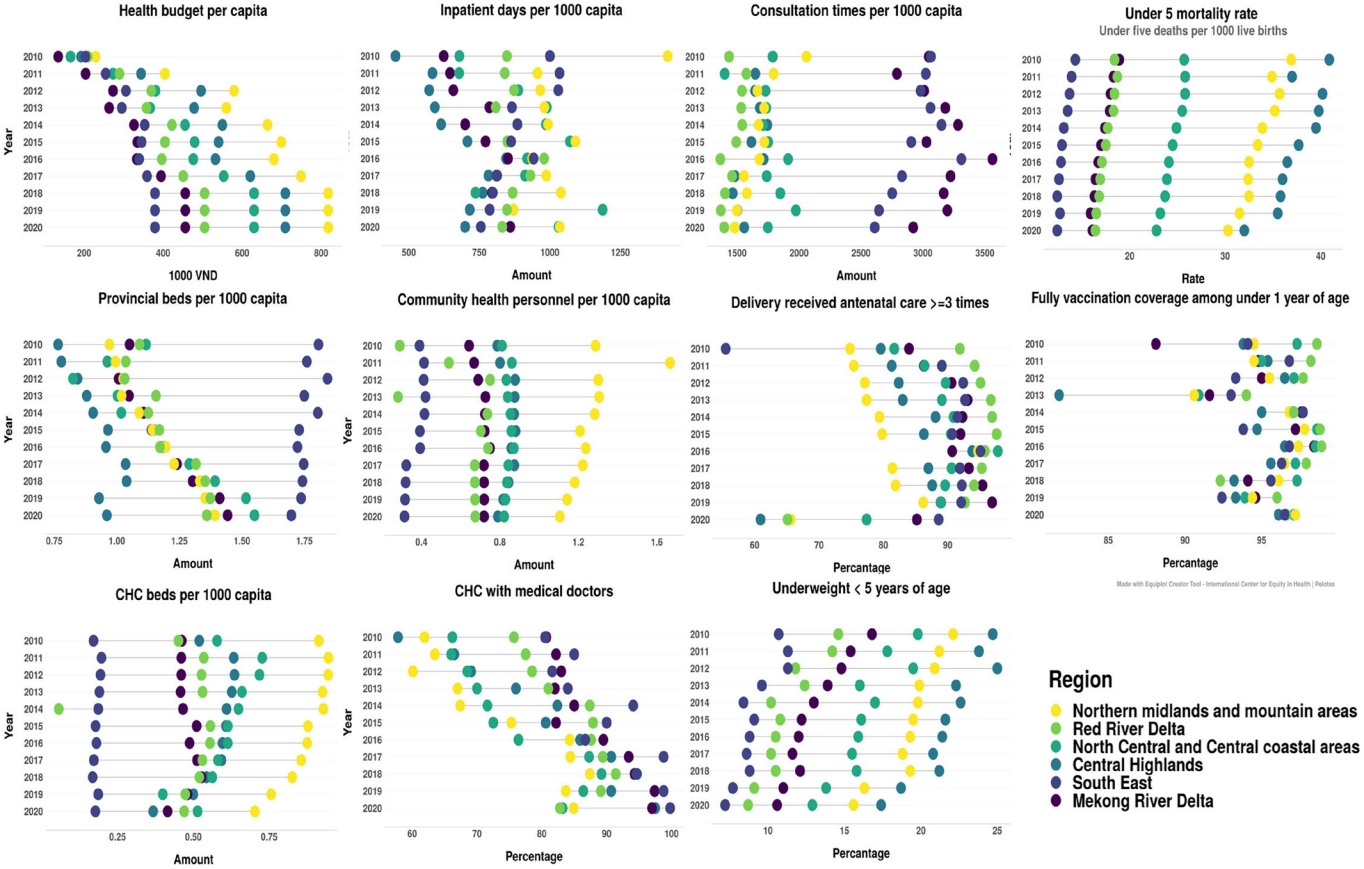
Regional inequalities in relation to several key indicators of Vietnam’s health system.

Changes in health indicators and equity based on income and incidence of poverty in Vietnam in recent years are shown in table 1. It can be seen that impoverished areas experienced unfavourable inequality in terms of the number of provincial-level hospital beds, delivery of antenatal care ≥3 times, the proportion of community health centres equipped with doctors, and mortality and malnutrition rates for children under 5 years of age. Figure 4 shows that although the absolute differences in the above indicators at the regional level are gradually narrowing (antenatal care coverage experienced a significant decrease in 2020 in the Red River Delta, Central Highlands, and Northern Midlands and Mountain Areas), the development of relatively disadvantaged indicators in Central Highlands, Northern Midlands and Mountain Areas, and South East is worth noting. We calculated the TI for these indicators, and the results are shown in online supplemental appendix table A6. The inequality in the malnutrition rate among children under 5 years of age comprised both intraregional and inter-regional differences, in similar proportions. Intraregional differences were the main contributors to inequality in antenatal care delivered ≥3 times, provincial hospital beds and community health service centres equipped with doctors. The Red River Delta and the South East region suffers from great intraregional differences in terms of malnutrition rate among children under 5 years of age, antenatal care delivered ≥3 times and community health service centres equipped with doctors.

**Table 1:** Overall development of health system indicator and health inequality in Vietnam

Dimension	Indicator	Overall development	Absolute inequality (SII)	Relative inequality (Concentration Index)	Specific performance
Health resources	Health budget	↑	●	●	Pro-poor
	Provincial hospital beds	↑	●		Pro-rich
	Hospital beds at community level	→	●	●	Pro-poor
	Total health personnel	↑	●	●	Pro-poor
	Health personnel at community level	→	●	●	Pro-poor
Health service delivery	Delivery received antenatal care ≥3 times	→	●	●	Pro-rich
	Full vaccination coverage among under 1 year of age	→			–
	Percentage of CHCs have medical doctor	↑	●	●	Pro-rich
Health service utilisation	Consultation times	→			–
	Inpatient days	↑			–
Health status	Under 5 mortality rate	↓	●	●	Pro-poor
	Underweight <5 years of age	↓	●	●	Pro-poor

Notes: ‘Overall Development’ refers to the development trend of the selected health indicators, not involving the judgement of equality. The up, horizontal and down arrows, respectively, represent the overall development level (amount, coverage and rate) showing an upward, unchanged and downward trend.

CHC, community health centre; SII, Slope Index of Inequality.

## Discussion

From 2010 to 2020, the overall level of health equity in Vietnam increased. The gap between affluent and poor provinces in terms of full immunisation for children was significantly reduced, and regional differences in the utilisation of health services (especially hospitalisation services) and full immunisation of children have continued to narrow. The per capita health budget, total health manpower, community hospital beds and community health manpower displayed significant pro-poor effects, and Vietnam’s health development strategy for poverty-stricken areas has been achieving its goals for a long time.

The Vietnamese population is experiencing rapid ageing, and the mortality rate from non-communicable diseases is gradually increasing,^35^ and thus the government has implemented reforms aimed at shifting the focus of healthcare from hospital-based services to primary healthcare, with primary healthcare facilities in suburban and rural areas being upgraded. However, inequality remains in terms of antenatal care and the proportion of community health service centres equipped with doctors. In terms of health status, Vietnam continues to display wealth-based inequality, which is disadvantageous for impoverished areas. Some researchers pointed out that Vietnam has long faced the problem of uneven geographical distribution of its healthcare workforce, with medical institutions in the large cities often providing better working conditions and development opportunities for health practitioners resulting in a lack of qualified primary healthcare personnel at the grass-roots level.^36^ Our findings indicate a good sign that the development of total health personnel and health personnel at community level shows a pro-poor trend. But such a sign does not mean everything is in good shape. Although inequality in terms of hardware (such as hospital beds) and total health workforce has been alleviated at the community level, significant inequalities remain in relation to the allocation of more professional health personnel (medical doctors) and service provision in impoverished areas. This may be related to the ongoing inequality in terms of health status.^37^

The issue of uneven regional health development continues to exist in Vietnam, as do local differences in mortality and malnutrition rates among children under 5 years of age. The Central Highlands, Northern Midlands and Mountain Areas, and North Central and Central Coastal Areas are at a relative disadvantage in terms of overall health development. Although the overall development level of the Red River Delta and the South East region is relatively high (Hanoi and Ho Chi Minh City are located in these regions), there are significant differences in relation to the development of the various provinces within these two regions, and it is possible that the large cities are siphoning off health resources from surrounding areas. In 2020, all regions’ antenatal care coverage experienced a decline. The disruption may be caused by COVID-19.^38^ Among the six regions, the Red River Delta, which had previously performed well in terms of this indicator, showed a sharp decline. This suggests that while paying attention to the resilience of the health system in underdeveloped areas, the government cannot ignore developed areas. The complex population structure and lack of primary healthcare levels in more developed areas can also have a significant impact on the provision of health services during special periods,^39^ which could cause a series of new inequity matters.

The current positive development trend should be maintained in the future. However, guaranteed access to affordable healthcare does not necessarily mean high-quality care.^40^ Health policymakers should pay more attention to the development of healthcare capacity in impoverished areas (especially provinces in the Central Highlands, Northern Midlands and Mountain Areas, and North Central and Central Coastal Areas), strengthen the attraction and training of medical talent (especially doctors at the community level) and enhance the ability to provide health services. Normally, the natural and socioeconomic conditions are much more difficult for undeveloped regions compared with other regions. So using innovative ways to alleviate the gap between affluent and poor areas in terms of quality of care and health status is essential,^41^ like developing mobile health services^17^ or trying to start national programmes to train qualified general practitioners with compulsory services in rural and remote areas.^42^ Adopting a transfer payment system for cross-subsidies, with developed areas subsidising underdeveloped areas, could also be helpful. Meanwhile, the Red River Delta and South East region should not be overlooked simply because of their relatively high levels of economic and healthcare development. The interruption of some basic public health services during the pandemic suggests that these regions also need to increase investment in primary healthcare to enhance the resilience of the health system. Further research is necessary to understand the causes of inequities in rapidly expanding and evolving regions in Vietnam,^43^ and it is also necessary to pay attention to the ongoing coordinated development within these areas when undertaking regional health planning. Multisector collaboration between national and subnational governments is necessary to achieve successful outcomes.^44^

A limitation of this study involves missing data (linear interpolation and other methods were used to fill in the gaps), which affected the results to some extent. Meanwhile, the highlight of this study is the use of official health statistics from Vietnam to comprehensively assess the development of health equity in Vietnam from 2010 to 2020 in four dimensions: health resources, health service delivery, health service utilisation and residents’ health status, thereby filling a gap in the literature regarding quantitative analysis of health equity in Vietnam. In the future, microdata should be used to examine the socioeconomic factors that are inhibiting the development of health equity in Vietnam, enabling targeted interventions to be implemented.

## Conclusion

Health equity in Vietnam has improved over the past decade, especially in the hardware aspect. However, inequities remain between affluent and poor areas in relation to the allocation of professional health workers and the provision of primary healthcare services. Health status varies among regions, and there are also variations among provinces within relatively developed regions. While it is necessary to strengthen investment in key areas and improve hardware, it is also important to focus on efficiency, strengthen service capabilities and adjust policies based on health outcomes. Vietnam’s health policies focused on the poor provide a lesson for other LMICs, as do the challenges faced by Vietnam in developing greater health equity.

## Data Availability

Data are available in a public, open access repository.
