# Supplementary material for: Progress towards health equity in Vietnam: evidence from nationwide official health statistics, 2010-2020
Source: BMJ Glob Health. 2024 Mar 18;9(3):e014739. doi: 10.1136/bmjgh-2023-014739 (PMC10952956; doi:10.1136/bmjgh-2023-014739)
Supplement: Supplementary data [file bmjgh-2023-014739supp001.pdf]

Appendix

Table A1 Health equity indicators

Dimension	Indicator
Health resources	Health budget per capita
	Provincial hospital beds per capita
	Per capita hospital beds at the community level
	Total health personnel per capita
	Health personnel at the community level per capita
Health service delivery	Delivery received antenatal care ⩾ 3 times
	Fully vaccination coverage < 1 year of age
	Percentage of CHCs with medical doctor
Health service utilization	Consultation times
	Inpatient days
Health status	Under 5 mortality rate
	Underweight < 5 years of age

Table A2 Trend testing of health system indicators in Vietnam (2010–2020)

Year	Health budget per capita (1000)	Prov. Beds (1000)	CHC Beds (1000)	Health personnell - total (1000)	Health personnel - community level (1000)	Consultation times (1000)	Inpatient days (1000)	Fully vac (%)	Delivery received antenatal care >= 3 times in 3 trimesters	% of CHCs have medical doctor	Underweight (%)	Under 5 mortality rate
**2010**	185.300	101.573	42.903	263.256	26.216	190876.500	72596.420	94.600	79.100	70.000	17.500	23.800
**2011**	281.050	97.255	49.470	279.797	67.999	180444.500	70185.890	96.000	86.700	71.900	16.800	23.300
**2012**	376.800	98.387	49.627	289.611	69.022	189684.200	75905.800	95.900	89.400	73.500	16.200	23.200
**2013**	364.000	105.012	48.700	305.147	27.591	196783.000	77351.620	91.400	90.000	75.000	15.300	23.100
**2014**	437.600	108.750	49.666	309.546	70.967	201662.900	78776.910	97.100	90.800	78.500	14.500	22.400
**2015**	443.100	114.615	49.544	318.373	70.143	195853.800	83656.950	97.200	90.600	79.800	14.100	22.100
**2016**	436.500	117.135	49.343	331.591	72.316	213820.200	85466.170	98.000	89.900	84.000	13.800	21.800
**2017**	494.700	126.952	48.892	330.516	69.481	198397.300	83113.860	96.800	90.600	89.100	13.400	21.500
**2018**	552.900	133.722	48.340	332.428	69.373	199384.200	79154.890	94.800	90.700	90.800	13.200	21.400
**2019**	552.900	139.696	42.874	334.340	69.265	201156.500	87486.880	96.800	91.200	89.200	13.400	21.000
**2020**	552.900	142.229	42.801	336.252	69.157	194109.700	86073.830	94.800	74.400	87.700	11.600	22.300
**β**	33.764	4.830	-0.279	7.131	2.781	1304.545	1495.928	0.115	-0.012	2.212	-0.515	-0.235
**p**	**0.000**	**0.000**	0.354	**0.000**	0.093	0.104	**0.000**	0.539	0.984	**0.000**	**0.000**	**0.001**

Table A3 SII values for key indicators of Vietnam's health system (2010–2020)

		Income sorting			Poverty rate sorting					Income sorting			Poverty rate sorting		
Indicator	Year	SII	95% CI: Upper-bound	95% CI: Lower-bound	SII	95% CI: Upper-bound	95% CI: Lower-bound	Indicator	Year	SII	95% CI: Upper-bound	95% CI: Lower-bound	SII	95% CI: Upper-bound	95% CI: Lower-bound
Per capita health budget	2010	-0.0857	0.4690	-0.6403	**0.8170**	1.4078	0.2262	Consultation times	2010	5.0696	12.3612	-2.2221	**-10.0081**	-2.0480	-17.9681
	2011	-0.0848	0.9997	-1.1694	**2.4859**	3.4400	1.5318		2011	4.8699	12.1031	-2.3632	**-13.1923**	-6.2055	-20.1791
	2012	**-4.1104**	-2.6107	-5.6101	**4.1746**	5.6136	2.7355		2012	7.9568	16.4746	-0.5610	**-9.4211**	-1.1987	-17.6435
	2013	**-3.9637**	-2.5060	-5.4214	**3.8933**	5.3074	2.4792		2013	**11.9400**	20.2676	3.6124	**-12.1834**	-4.1251	-20.2416
	2014	**-5.4294**	-3.5127	-7.3461	**4.7601**	6.6758	2.8444		2014	**9.7851**	18.6250	0.9452	**-11.1266**	-2.7889	-19.4641
	2015	**-5.2676**	-3.6200	-6.9153	**4.9129**	6.4905	3.3354		2015	5.9224	13.7136	-1.8688	-6.8661	0.4469	-14.1790
	2016	**-5.0478**	-3.4819	-6.6136	**4.7804**	6.2930	3.2677		2016	3.1818	16.2794	-9.9158	-7.8096	4.6073	-20.2266
	2017	**-5.5287**	-3.9963	-7.0610	**5.3947**	6.8829	3.9065		2017	3.4822	13.0584	-6.0939	-2.0965	7.2490	-11.4420
	2018	**-5.8040**	-4.0916	-7.5164	**6.1570**	7.8286	4.4855		2018	3.5721	12.6735	-5.5292	-2.8437	6.3972	-12.0847
	2019	**-5.8915**	-4.1853	-7.5977	**6.1507**	7.8407	4.4607		2019	3.5200	12.5922	-5.5522	-2.6811	6.5408	-11.9030
	2020	**-5.9914**	-4.2691	-7.7137	**6.3595**	8.0197	4.6993		2020	2.3446	10.0038	-5.3146	-2.6797	5.0356	-10.3951
Per capita provincial hospital beds	2010	-0.0017	0.3450	-0.3484	**-0.4183**	-0.0418	-0.7949	Inpatient days	2010	2.1958	20.5355	-16.1440	**-38.6899**	-20.4947	-56.8851
	2011	0.0122	0.3584	-0.3339	**-0.5229**	-0.1823	-0.8636		2011	-3.5183	16.4147	-23.4513	**-35.7651**	-16.7594	-54.7708
	2012	**0.5680**	0.9575	0.1784	**-0.5696**	-0.1898	-0.9495		2012	**35.0804**	53.6490	16.5118	**-35.6903**	-17.6962	-53.6844
	2013	**0.5021**	0.8515	0.1527	**-0.5488**	-0.2145	-0.8831		2013	**29.9086**	48.1142	11.7030	**-30.6098**	-13.0401	-48.1794
	2014	**0.5834**	0.9516	0.2151	**-0.5947**	-0.2454	-0.9441		2014	**37.5575**	55.7228	19.3922	**-34.3629**	-34.3629	-52.0000
	2015	**0.5856**	1.0078	0.1634	**-0.5415**	-0.1402	-0.9427		2015	**23.8906**	42.0165	5.7647	**-23.2379**	-6.1149	-40.3609
	2016	**0.5237**	0.9674	0.0800	**-0.5322**	-0.1094	-0.9550		2016	**17.7257**	31.9218	3.5296	**-16.0671**	-2.3885	-29.7456
	2017	**0.5064**	0.9487	0.0642	**-0.5092**	-0.0800	-0.9384		2017	**16.2404**	28.6198	3.8609	**-18.4678**	-6.6694	-30.2662
	2018	0.5104	1.0313	-0.0106	**-0.5664**	-0.0418	-1.0910		2018	8.6378	20.0088	-2.7332	-10.8640	0.5408	-22.2688
	2019	0.5270	1.0634	-0.0095	**-0.5531**	-0.0102	-1.0960		2019	**16.0253**	26.8684	5.1822	**-16.4976**	-5.5228	-27.4724
	2020	0.4488	0.9893	-0.0917	-0.3771	0.1716	-0.9258		2020	-1.6958	10.2215	-13.6130	0.1187	-11.9045	12.1418
Per capita hospital beds at community level	2010	-0.1228	0.1520	-0.3977	**0.7758**	1.0169	0.5347	CHCs have medical doctor	2010	2.1958	20.5355	-16.1440	**-38.6899**	-20.4947	-56.8851
	2011	**-0.3932**	-0.1020	-0.6844	**0.8151**	1.0652	0.5649		2011	-3.5183	16.4147	-23.4513	**-35.7651**	-16.7594	-54.7708
	2012	**-0.8374**	-0.5947	-1.0801	**0.7918**	1.0353	0.5484		2012	**35.0804**	53.6490	16.5118	**-35.6903**	-17.6962	-53.6844
	2013	**-0.7653**	-0.5092	-1.0215	**0.6975**	0.9559	0.4390		2013	**29.9086**	48.1142	11.7030	**-30.6098**	-13.0401	-48.1794
	2014	**-0.7376**	-0.4761	-0.9991	**0.6641**	0.9225	0.4057		2014	**37.5575**	55.7228	19.3922	**-34.3629**	-34.3629	-52.0000
	2015	**-0.6466**	-0.3990	-0.8942	**0.5773**	0.8177	0.3369		2015	**23.8906**	42.0165	5.7647	**-23.2379**	-6.1149	-40.3609
	2016	**-0.5959**	-0.3366	-0.8551	**0.5880**	0.8338	0.3421		2016	**17.7257**	31.9218	3.5296	**-16.0671**	-2.3885	-29.7456
	2017	**-0.6161**	-0.3650	-0.8672	**0.5476**	0.7998	0.2953		2017	**16.2404**	28.6198	3.8609	**-18.4678**	-6.6694	-30.2662
	2018	**-0.5397**	-0.2955	-0.7840	**0.5101**	0.7627	0.2574		2018	8.6378	20.0088	-2.7332	-10.8640	0.5408	-22.2688
	2019	**-0.5550**	-0.3146	-0.7954	**0.5389**	0.7862	0.2915		2019	**16.0253**	26.8684	5.1822	**-16.4976**	-5.5228	-27.4724
	2020	**-0.5029**	-0.2406	-0.7651	**0.5307**	0.7920	0.2694		2020	-1.6958	10.2215	-13.6130	0.1187	-11.9045	12.1418
Per capita total health personnel	2010	0.0775	-0.7623	0.9174	**2.1661**	2.9356	1.3966	Delivery received antenatal care >=3 times	2010	-8.1588	4.1906	-20.5082	**-20.4214**	-7.2818	-33.5609
	2011	-0.1537	-1.0561	0.7486	**2.0745**	2.8681	1.2808		2011	-10.4445	1.5801	-22.4691	**-29.4959**	-18.8847	-40.1072
	2012	**-2.0811**	-2.9387	-1.2235	**2.0621**	2.8970	1.2272		2012	**30.6674**	40.6850	20.6498	**-30.2887**	-20.5447	-40.0327
	2013	**-2.1755**	-3.0670	-1.2839	**2.0563**	2.9346	1.1780		2013	**29.6363**	38.9298	20.3427	**-30.0229**	-21.2201	-38.8257
	2014	**-2.0292**	-1.1821	-2.8763	**1.8593**	2.6849	1.0338		2014	**27.6542**	36.6910	18.6173	**-25.3830**	-16.4971	-34.2690
	2015	**-1.7957**	-0.9191	-2.6723	**1.6569**	2.4935	0.8203		2015	**29.1149**	38.0047	20.2250	**-27.5513**	-19.1195	-35.9830
	2016	**-1.8852**	-1.0311	-2.7394	**1.8183**	2.6353	1.0013		2016	**21.1300**	32.1608	10.0993	**-19.9373**	-9.3265	-30.5480
	2017	**-1.7463**	-0.9077	-2.5850	**1.8088**	2.6095	1.0080		2017	**26.5034**	36.5584	16.4484	**-25.5564**	-15.7287	-35.3842
	2018	**-1.4612**	-0.6177	-2.3047	**1.5404**	2.3883	0.6926		2018	**19.7925**	29.5269	10.0581	**-22.1878**	-12.6268	-31.7489
	2019	**-1.2356**	-0.2776	-2.1936	**1.2295**	2.2032	0.2558		2019	**15.2552**	24.9158	5.5946	**-16.6890**	-7.0215	-26.3564
	2020	**-1.1491**	-0.0188	-2.2794	0.9928	2.1426	-0.1571		2020	**31.2908**	50.8941	11.6875	**-25.6190**	-5.3003	-45.9378
Per capita health personnel at community level	2010	-0.0063	0.2853	-0.2979	**1.0280**	1.2258	0.8301	Fully vaccination coverage among under 1 year of age	2010	0.8972	5.8956	-4.1011	-1.8905	3.7383	-7.5192
	2011	-0.1680	0.1237	-0.4598	**0.9130**	1.1194	0.7066		2011	1.0678	4.1615	-2.0258	-2.6588	0.5535	-5.8711
	2012	**-0.9856**	-0.7699	-1.2012	**0.9636**	1.1748	0.7525		2012	-0.5699	2.9025	-4.0423	0.9260	4.3182	-2.4661
	2013	**-1.0253**	-0.8111	-1.2395	**0.9655**	1.1841	0.7469		2013	5.0824	10.9748	-0.8099	**-6.3562**	-0.7082	-12.0043
	2014	**-0.9991**	-0.7898	-1.2083	**0.9401**	1.1463	0.7339		2014	2.6037	4.9810	0.2265	**-3.1321**	-0.9045	-5.3598
	2015	**-0.9779**	-0.7732	-1.1826	**0.9283**	1.1217	0.7349		2015	-0.7810	1.1369	-2.6990	0.3833	2.2079	-1.4413
	2016	**-0.9853**	-0.7357	-1.2349	**0.9648**	1.1985	0.7310		2016	**2.0379**	3.4154	0.6604	**-1.7778**	-0.4415	-3.1141
	2017	**-1.0317**	-0.8145	-1.2489	**0.9833**	1.2010	0.7655		2017	0.9799	3.2993	-1.3395	-1.5203	0.7168	-3.7573
	2018	**-1.0192**	-0.6977	-1.3408	**1.0255**	1.3529	0.6981		2018	0.5201	3.6566	-2.6165	-1.5464	1.6103	-4.7030
	2019	**-1.0508**	-0.4865	-1.6151	**1.0447**	1.6197	0.4698		2019	1.8160	5.9911	-2.3591	-2.7788	1.4225	-6.9801
	2020	**-1.2022**	-0.3765	-2.0280	**1.0579**	1.9041	0.2116		2020	0.6483	2.3453	-1.0487	-0.9485	0.7534	-2.6504
Underweight < 5 years of age	2010	-0.2065	3.5683	-3.9814	**14.3485**	16.5104	12.1865	Under 5 mortality rate	2010	4.422026	15.04768	-6.203632	**34.70763**	42.86885	26.54641
	2011	-2.6566	1.3839	-6.6972	**14.7036**	16.8474	12.5598		2011	-0.0453805	11.36159	-11.45235	**34.56138**	42.75638	26.36638
	2012	**-13.6761**	-11.1379	-16.2142	**14.3093**	16.4263	12.1924		2012	**-32.94545**	-23.88325	-42.00765	**34.31358**	42.65288	25.97428
	2013	**-14.3115**	-11.7119	-16.9110	**14.7838**	16.9801	12.5875		2013	**-32.7579**	-23.6884	-41.8274	**33.78241**	42.14757	25.41726
	2014	**-14.6485**	-12.2468	-17.0503	**14.4831**	16.6019	12.3643		2014	**-32.74167**	-24.07751	-41.40582	**32.27756**	40.36196	24.19315
	2015	**-14.7316**	-12.3207	-17.1424	**14.3431**	16.4660	12.2202		2015	**-32.20886**	-23.7624	-40.65532	**31.03545**	38.90817	23.16274
	2016	**-14.6200**	-12.3338	-16.9063	**14.1508**	16.2825	12.0191		2016	**-31.23309**	-23.0323	-39.43389	**30.40113**	38.14217	22.6601
	2017	**-14.5924**	-11.8492	-17.3356	**14.8638**	17.2866	12.4410		2017	**-30.95156**	-22.84308	-39.06003	**31.39798**	38.95799	23.83797
	2018	**-13.7897**	-11.5253	-16.0541	**14.3454**	16.4848	12.2061		2018	**-30.35893**	-22.6153	-38.10256	**31.74287**	39.33061	24.15513
	2019	**-13.1220**	-11.2557	-14.9884	**13.4276**	15.2655	11.5897		2019	**-30.19959**	-22.65008	-37.7491	**31.18174**	38.69263	23.67084
	2020	**-12.4289**	-10.5855	-14.2723	**12.9492**	14.6094	11.2891		2020	**-29.2839**	-22.46339	-36.10441	**29.56821**	36.43254	22.70388

***Bold SII represents P<0.05**

Table A4 Results of trend testing of the SII and CI

Indicator	Index	Sorting	β	p
Per capita health budget	SII	Income	-0.551	**0.001**
Per capita health budget	SII	Poverty rate	0.467	**<0.001**
Per capita provincial hospital beds	SII	Income	0.037	0.066
Per capita provincial hospital beds	SII	Poverty rate	0.002	0.752
Per capita hospital beds at community level	SII	Income	-0.011	0.582
Per capita hospital beds at community level	SII	Poverty rate	-0.032	**<0.001**
Per capita total health personnel	SII	Income	-0.069	0.369
Per capita total health personnel	SII	Poverty rate	-0.103	**<0.001**
Per capita health personnel at community level	SII	Income	-0.087	**0.008**
Per capita health personnel at community level	SII	Poverty rate	0.008	0.068
Underweight < 5 years of age	SII	Income	-0.944	**0.048**
Underweight < 5 years of age	SII	Poverty rate	-0.111	**0.036**
Consultation times	SII	Income	-0.506	0.080
Consultation times	SII	Poverty rate	1.108	**<0.001**
Inpatient days	SII	Income	-0.616	0.673
Inpatient days	SII	Poverty rate	3.529	**<0.001**
CHCs have medical doctor	SII	Income	-0.616	0.673
CHCs have medical doctor	SII	Poverty rate	3.529	**<0.001**
Delivery received antenatal care >=3 times	SII	Income	2.315	0.108
Delivery received antenatal care >=3 times	SII	Poverty rate	0.581	0.191
Fully vaccination coverage among under 1 year of age	SII	Income	-0.034	0.838
Fully vaccination coverage among under 1 year of age	SII	Poverty rate	0.071	0.719
Under 5 mortality rate	SII	Income	-2.512	**0.047**
Under 5 mortality rate	SII	Poverty rate	-0.487	**<0.001**
Per capita health budget	CI	Income	-0.011	**0.048**
Per capita health budget	CI	Poverty rate	0.004	0.145
Per capita provincial hospital beds	CI	Income	0.003	0.215
Per capita provincial hospital beds	CI	Poverty rate	0.002	0.052
Per capita hospital beds at community level	CI	Income	-0.003	0.484
Per capita hospital beds at community level	CI	Poverty rate	-0.008	**<0.001**
Per capita total health personnel	CI	Income	-0.002	0.472
Per capita total health personnel	CI	Poverty rate	-0.006	**<0.001**
Per capita health personnel at community level	CI	Income	-0.014	**0.012**
Per capita health personnel at community level	CI	Poverty rate	-0.001	0.327
Underweight < 5 years of age	CI	Income	-0.012	**0.007**
Underweight < 5 years of age	CI	Poverty rate	0.003	**0.001**
Consultation times	CI	Income	-0.004	**0.045**
Consultation times	CI	Poverty rate	0.008	**<0.001**
Inpatient days	CI	Income	-0.006	0.109
Inpatient days	CI	Poverty rate	0.000	0.910
CHCs have medical doctor	CI	Income	-0.002	0.529
CHCs have medical doctor	CI	Poverty rate	0.009	**<0.001**
Delivery received antenatal care >=3 times	CI	Income	0.005	0.080
Delivery received antenatal care >=3 times	CI	Poverty rate	0.001	0.108
Fully vaccination coverage among under 1 year of age	CI	Income	0.000	0.904
Fully vaccination coverage among under 1 year of age	CI	Poverty rate	0.000	0.720
Under 5 mortality rate	CI	Income	-0.018	**0.019**
Under 5 mortality rate	CI	Poverty rate	-0.001	**0.028**

Table A5 CI values for key indicators of Vietnam's health system (2010–2020)

		Income sorting		Poverty rate sorting				Income sorting		Poverty rate sorting	
**Indicator**	**Year**	**CI**	**p-value**	**CI**	**p-value**	**Indicator**	**Year**	**CI**	**p-value**	**CI**	**p-value**
**Per capita health budget**	2010	-0.0094	0.7229	**0.0763**	0.0028	**Consultation times**	2010	0.0414	0.1706	**-0.0661**	0.0266
	2011	-0.0086	0.7698	**0.1380**	0.0000		2011	0.0447	0.1477	**-0.1026**	0.0005
	2012	**-0.1570**	0.0000	**0.1649**	0.0000		2012	0.0570	0.0806	**-0.0712**	0.0279
	2013	**-0.1566**	0.0000	**0.1613**	0.0000		2013	**0.0845**	0.0067	**-0.0882**	0.0046
	2014	**-0.1693**	0.0000	**0.1595**	0.0000		2014	0.0668	0.0342	**-0.0791**	0.0115
	2015	**-0.1620**	0.0000	**0.1652**	0.0000		2015	0.0402	0.1499	-0.0506	0.0683
	2016	**-0.1600**	0.0000	**0.1624**	0.0000		2016	0.0217	0.6199	-0.0541	0.2131
	2017	**-0.1522**	0.0000	**0.1551**	0.0000		2017	0.0248	0.4659	-0.0148	0.6650
	2018	**-0.1457**	0.0000	**0.1518**	0.0000		2018	0.0285	0.3994	-0.0233	0.4899
	2019	**-0.1470**	0.0000	**0.1509**	0.0000		2019	0.0288	0.3878	-0.0221	0.5091
	2020	**-0.1493**	0.0000	**0.1549**	0.0000		2020	0.0213	0.4685	-0.0239	0.4154
**Per capita provincial hospital beds**	2010	-0.0008	0.9783	-0.0529	0.0561	**Inpatient days**	2010	0.0903	0.2704	0.0707	0.3888
	2011	-0.0004	0.9884	**-0.0709**	0.0094		2011	-0.0232	0.4941	-0.0197	0.5618
	2012	**0.0778**	0.0089	**-0.0775**	0.0092		2012	0.0020	0.9431	0.0282	0.6523
	2013	**0.0665**	0.0083	**-0.0713**	0.0045		2013	-0.0087	0.7767	-0.0059	0.8471
	2014	**0.0739**	0.0041	**-0.0746**	0.0037		2014	0.0117	0.6975	-0.0081	0.7876
	2015	**0.0698**	0.0110	**-0.0658**	0.0167		2015	-0.0409	0.0776	0.0341	0.1432
	2016	0.0450	0.1329	-0.0526	0.0783		2016	-0.0080	0.8203	-0.0112	0.7494
	2017	0.0567	0.0308	**-0.0555**	0.0348		2017	-0.0460	0.0958	0.0378	0.1720
	2018	0.0577	0.0516	**-0.0604**	0.0415		2018	-0.0252	0.3440	0.0297	0.2646
	2019	0.0571	0.0535	-0.0571	0.0534		2019	0.0154	0.6201	-0.0063	0.8405
	2020	0.0479	0.0953	-0.0388	0.1776		2020	-0.0337	0.1932	0.0498	0.0522
**Per capita hospital beds at community level**	2010	-0.0352	0.3909	**0.2012**	0.0000	**CHCs have medical doctor**	2010	0.0062	0.7943	**-0.0922**	0.0000
	2011	**-0.0973**	0.0087	**0.1879**	0.0000		2011	-0.0084	0.7190	**-0.0832**	0.0001
	2012	**-0.1903**	0.0000	**0.1820**	0.0000		2012	**0.0759**	0.0002	**-0.0804**	0.0001
	2013	**-0.1767**	0.0000	**0.1654**	0.0000		2013	**0.0628**	0.0012	**-0.0666**	0.0006
	2014	**-0.1647**	0.0000	**0.1544**	0.0000		2014	**0.0728**	0.0001	**-0.0709**	0.0001
	2015	**-0.1441**	0.0000	**0.1356**	0.0000		2015	**0.0457**	0.0087	**-0.0472**	0.0066
	2016	**-0.1363**	0.0000	**0.1396**	0.0000		2016	**0.0319**	0.0140	**-0.0293**	0.0246
	2017	**-0.1405**	0.0000	**0.1294**	0.0001		2017	**0.0269**	0.0106	**-0.0312**	0.0027
	2018	**-0.1307**	0.0001	**0.1236**	0.0002		2018	0.0140	0.1445	**-0.0175**	0.0665
	2019	**-0.1364**	0.0000	**0.1323**	0.0001		2019	**0.0260**	0.0057	**-0.0274**	0.0035
	2020	**-0.1251**	0.0005	**0.1323**	0.0002		2020	-0.0032	0.7398	0.0001	0.9900
**Per capita total health personnel**	2010	0.0039	0.8623	**0.1088**	0.0000	**Delivery received antenatal care >=3 times**	2010	-0.0184	0.1638	**-0.0444**	0.0005
	2011	-0.0069	0.7485	**0.0995**	0.0000		2011	-0.0186	0.1140	**-0.0584**	0.0000
	2012	**-0.0943**	0.0000	**0.0965**	0.0000		2012	**0.0560**	0.0000	**-0.0570**	0.0000
	2013	**-0.0934**	0.0000	**0.0926**	0.0000		2013	**0.0531**	0.0000	**-0.0559**	0.0000
	2014	**-0.0835**	0.0000	**0.0833**	0.0000		2014	**0.0483**	0.0000	**-0.0474**	0.0000
	2015	**-0.0731**	0.0001	**0.0742**	0.0000		2015	**0.0503**	0.0000	**-0.0517**	0.0000
	2016	**-0.0759**	0.0000	**0.0785**	0.0000		2016	**0.0371**	0.0001	**-0.0369**	0.0002
	2017	**-0.0712**	0.0000	**0.0777**	0.0000		2017	**0.0467**	0.0000	**-0.0466**	0.0000
	2018	**-0.0606**	0.0007	**0.0637**	0.0003		2018	**0.0364**	0.0000	**-0.0397**	0.0000
	2019	**-0.0504**	0.0111	**0.0499**	0.0120		2019	**0.0271**	0.0011	**-0.0295**	0.0003
	2020	-0.0444	0.0538	0.0378	0.1022		2020	**0.0646**	0.0013	**-0.0528**	0.0097
**Per capita health personnel at community level**	2010	-0.0013	0.9656	**0.1873**	0.0000	**Fully vaccination coverage among under 1 year of age**	2010	0.0020	0.6652	-0.0035	0.4539
	2011	-0.0313	0.2461	**0.1601**	0.0000		2011	0.0016	0.5416	-0.0043	0.1056
	2012	**-0.1666**	0.0000	**0.1658**	0.0000		2012	-0.0007	0.8114	0.0012	0.6709
	2013	**-0.1712**	0.0000	**0.1651**	0.0000		2013	0.0086	0.0838	**-0.0110**	0.0256
	2014	**-0.1638**	0.0000	**0.1615**	0.0000		2014	**0.0043**	0.0217	**-0.0053**	0.0040
	2015	**-0.1622**	0.0000	**0.1640**	0.0000		2015	-0.0009	0.5574	0.0000	0.9800
	2016	**-0.1653**	0.0000	**0.1697**	0.0000		2016	0.0035	0.0013	**-0.0032**	0.0029
	2017	**-0.1753**	0.0000	**0.1729**	0.0000		2017	0.0017	0.3512	-0.0027	0.1350
	2018	**-0.1721**	0.0000	**0.1706**	0.0000		2018	0.0013	0.6074	-0.0028	0.2635
	2019	**-0.1699**	0.0005	**0.1639**	0.0008		2019	0.0036	0.2768	-0.0050	0.1324
	2020	**-0.1871**	0.0053	**0.1595**	0.0186		2020	0.0014	0.2956	-0.0017	0.2037
**Underweight < 5 years of age**	2010	**-0.0009**	0.9594	**0.1165**	0.0000	**Under 5 mortality rate**	2010	0.0316	0.3719	**0.2108**	0.0000
	2011	**-0.0259**	0.1764	**0.1288**	0.0000		2011	-0.0023	0.9491	**0.2110**	0.0000
	2012	-0.1218	0.0000	**0.1298**	0.0000		2012	**-0.1945**	0.0000	**0.2086**	0.0000
	2013	-0.1346	0.0000	**0.1429**	0.0000		2013	**-0.1942**	0.0000	**0.2080**	0.0000
	2014	-0.1461	0.0000	**0.1505**	0.0000		2014	**-0.1982**	0.0000	**0.2061**	0.0000
	2015	-0.1485	0.0000	**0.1525**	0.0000		2015	**-0.1971**	0.0000	**0.2030**	0.0000
	2016	-0.1524	0.0000	**0.1524**	0.0000		2016	**-0.1964**	0.0000	**0.2015**	0.0000
	2017	-0.1510	0.0000	**0.1578**	0.0000		2017	**-0.1965**	0.0000	**0.2078**	0.0000
	2018	-0.1524	0.0000	**0.1562**	0.0000		2018	**-0.2011**	0.0000	**0.2082**	0.0000
	2019	-0.1497	0.0000	**0.1513**	0.0000		2019	**-0.2021**	0.0000	**0.2068**	0.0000
	2020	-0.1478	0.0000	**0.1521**	0.0000		2020	**-0.1980**	0.0000	**0.1976**	0.0000

Feng Y, *et al. BMJ Glob Health* 2024; 9:e014739. doi: 10.1136/bmjgh-2023-014739

Table A6 Theil index values for several key indicators of Vietnam's health system (2010–2020)

Indicator	Year	TWR	TBR	Theil	Central highlands	Mekong river delta	North central and central coastal areas	Northern midlands and mountain areas	Red river delta	South east
Underweight (<5 years old)	2010	0.1798081	0.1274555	0.3072636	0.1872525	0.048258	0.1377031	0.1448882	0.2879025	0.5584376
	2011	0.1822025	0.1321315	0.314334	0.1900745	0.0502584	0.1411432	0.1361673	0.3133782	0.5668976
	2012	0.1840693	0.134264	0.3183333	0.1865702	0.0507889	0.1425911	0.1356107	0.3204904	0.5866106
	2013	0.1886935	0.1464244	0.3351179	0.1912114	0.0580034	0.1433152	0.1362314	0.328438	0.6302799
	2014	0.186851	0.1519573	0.3388083	0.185464	0.0554868	0.1489868	0.1397771	0.3403115	0.5709009
	2015	0.1901315	0.1578681	0.3479995	0.1817494	0.059434	0.1555755	0.1429571	0.3451853	0.5751589
	2016	0.1873385	0.1558033	0.3431419	0.178984	0.0573138	0.1490441	0.1385759	0.3493628	0.5787966
	2017	0.1891492	0.1663903	0.3555395	0.1079033	0.0626307	0.181588	0.1451757	0.3515606	0.5821647
	2018	0.1924049	0.1618862	0.3542911	0.1654266	0.0629182	0.159411	0.1455368	0.3548376	0.5832384
	2019	0.1921953	0.1591772	0.3513724	0.155873	0.0609669	0.1554386	0.1473452	0.3539639	0.6133122
	2020	0.1918079	0.1615366	0.3533445	0.1563631	0.0577227	0.1543031	0.1505322	0.3520108	0.612155
% of delivery received antenatal care >=3 times in 3 trimesters	2010	0.1696609	0.0413668	0.2110277	0.0845445	0.0677345	0.147206	0.0757977	0.2238528	0.6130573
	2011	0.1399942	0.0343375	0.1743317	0.0835858	0.060465	0.1351912	0.068284	0.2202892	0.3426137
	2012	0.1359804	0.0338816	0.169862	0.0802784	0.0516306	0.1254521	0.0715401	0.2204128	0.3385853
	2013	0.1415004	0.0355573	0.1770577	0.1023537	0.045941	0.1392428	0.0819089	0.2204107	0.342544
	2014	0.1397121	0.039199	0.1789111	0.0921293	0.0379205	0.1413376	0.0804331	0.2212002	0.3492458
	2015	0.141241	0.04231	0.1835511	0.0902095	0.0473509	0.151112	0.0700113	0.2175803	0.3515099
	2016	0.143277	0.0405265	0.1838036	0.1635284	0.0436975	0.1336678	0.0904941	0.2253398	0.3113235
	2017	0.1422217	0.0478726	0.1900943	0.1052793	0.0382583	0.1489217	0.085626	0.2139508	0.3584219
	2018	0.1450823	0.0518707	0.1969531	0.1096308	0.04132	0.1480012	0.0954234	0.2180124	0.3610972

Feng Y, *et al. BMJ Glob Health* 2024; 9:e014739. doi: 10.1136/bmjgh-2023-014739

	2019	0.1480223	0.0598816	0.2079038	0.1106266	0.0397261	0.1660686	0.0999397	0.2156397	0.36013
	2020	0.1833843	0.0527693	0.2361536	0.1560868	0.0525881	0.1720233	0.1426235	0.316016	0.3749613
**Provincial hospital beds**	2010	0.0652843	0.029027	0.0943112	0.0576365	0.0337306	0.0767406	0.0380359	0.0467224	0.1051056
	2011	0.0665444	0.0327955	0.0993398	0.0378746	0.0262141	0.1159375	0.0327059	0.0305911	0.1050622
	2012	0.1037234	0.0425308	0.1462542	0.0290103	0.0299902	0.32028	0.0226102	0.0595309	0.1061302
	2013	0.0569135	0.0248773	0.0817907	0.0232608	0.0279769	0.0874186	0.0266567	0.0343962	0.0934044
	2014	0.0604202	0.0253011	0.0857213	0.0192794	0.0284262	0.0906997	0.0244818	0.0491905	0.0935406
	2015	0.0660528	0.0159589	0.0820117	0.0218766	0.0256184	0.1156096	0.0238161	0.0536483	0.094218
	2016	0.0683912	0.014106	0.0824972	0.0231983	0.0402741	0.1240381	0.0297663	0.0511458	0.0860158
	2017	0.059168	0.0104468	0.0696148	0.0192246	0.0335472	0.0852584	0.0226063	0.0428873	0.097091
	2018	0.0687349	0.0080385	0.0767734	0.0354173	0.0333037	0.1206901	0.0298331	0.0451214	0.0982222
	2019	0.0670274	0.0088344	0.0758618	0.0551288	0.0300854	0.116285	0.02765	0.0521718	0.0858617
	2020	0.0633871	0.0075428	0.0709299	0.0726905	0.0281161	0.0928531	0.0350619	0.0522932	0.087362
**% of CHCs with medical doctor**	2010	0.1241103	0.0327658	0.1568761	0.0415251	0.0540315	0.1364	0.0736684	0.2033067	0.2523349
	2011	0.1403762	0.0305901	0.1709663	0.151157	0.0659162	0.1500433	0.0838332	0.2008231	0.2647407
	2012	0.1364115	0.0334492	0.1698607	0.1518434	0.0585364	0.1493969	0.0797574	0.1839636	0.2851559
	2013	0.1469895	0.0365939	0.1835833	0.1585815	0.0628349	0.1735747	0.070295	0.1917202	0.314853
	2014	0.1579189	0.0315308	0.1894497	0.1349819	0.0671943	0.1684435	0.1353587	0.1888734	0.3177361
	2015	0.1454646	0.0432952	0.1887598	0.1346273	0.0590197	0.169326	0.0740817	0.1959493	0.3218783
	2016	0.1416208	0.0523442	0.193965	0.1157584	0.0694634	0.1623382	0.0665645	0.2008038	0.3276087
	2017	0.1455143	0.0525865	0.1981008	0.1400899	0.053777	0.1540234	0.0889728	0.2106098	0.3233055
	2018	0.1471921	0.0598902	0.2070823	0.1302688	0.0631836	0.1556965	0.0884886	0.2342595	0.2959281
	2019	0.1463214	0.0567755	0.203097	0.1357977	0.0397866	0.1519232	0.1020811	0.2247113	0.3281222
	2020	0.1512688	0.0679106	0.2191794	0.1213968	0.0401607	0.1615409	0.0993319	0.2519045	0.3413826

Feng Y, *et al. BMJ Glob Health* 2024; 9:e014739. doi: 10.1136/bmjgh-2023-014739
